# 

**Published:** 1914-11-14

**Authors:** 


### THERMOGENE.

We have been informed, and are asked to state, that "Thermogene was invented by Charles Vandenbroeck, of Brussels, the Belgian chemist, from whom it was acquired by the present British proprietors fourteen years ago. Thermogene is entirely British-owned, and is British-made by British labour at the company's factories at Hayward's Heath, Sussex. It is used by the British Red Cross Society and the Royal Navy."

### Medical Appointments.

Mr. H. M. Rashbrook, fifth assistant medical officer at Claybury Mental Hospital, has been appointed third assistant medical officer; and Mr. T. Fennessy has been appointed fourth assistant medical officer.

The following have been appointed by the Metropolitan Asylums Board assistant medical officers :—Drs. C. Aldis, J. C. H. Allan, A. C. Anderson, C. M. G. Campbell, J. H. Coats, J. Connell, P. Figdor, H. Fleming, J. H. Gibbons, M. Gross, E. B. Gunson, E. L. Horsburgh, W. Forsyth Jones, W. B. Lawrence, O. C. Link, R. Swyer, and R. H. M. Tauffe.

The following appointments have been made at the Royal Free Hospital : Acting assistant surgeons, Henry Blakeway, F.R.C.S., and Harold Gardiner, F.R.C.S.; resident house physicians, Miss M. Lloyd, M.B., B.S., and Miss M. R. Paterson, M.R.C.S., L.R.C.P.; resident house surgeons, Miss M. E. G. Pillman, M.R.C.S., L.R.C.P., and Miss E. M. Powell, M.R.C.S., L.R.C.P.; senior clinical assistant, gynæcological department, Miss A. Blair, M.B., B.S.; and clinical assistant, skin department, Mrs. Addison, M.B., B.S.

### Honour for a Wardsman.

Private W. A. Flaxman, R.A.M.C., who was an assistant wardsman at the Southwark Infirmary, was mentioned in dispatches from the General Headquarters Staff on October 8. He was trained at Netley Hospital. The Southwark Board of Guardians have decided to send to Private Flaxman a letter of commendation for his bravery on the battlefield, coupled with their hearty good wishes for his future success.

### Buildings Contemplated.

Paignton.—The Urban District Council have received the sanction of the Local Government Board to a loan of £2,500 for the extension of the isolation hospital.

Penn.—The Local Government Board have approved the site at Penn for the sanatorium to be built by the Staffordshire, Wolverhampton, and Dudley Joint Tuberculosis Committee.

St. Austell.—The Guardians are negotiating for a loan of £3,986 for infirmary reconstruction.

Tynemouth.—The Corporation Health Committee have approved the sketched plans, submitted to them by the borough surveyor, of an isolation hospital with sixty beds and a sanatorium with twenty-four beds, and have ordered them to be submitted to the Local Government Board. The estimated cost would be £30,000 for the hospital and £2,700 for the sanatorium.

### The Book Market.

LIST OF NEW BOOKS RECEIVED FROM THE FOLLOWING PUBLISHERS:—

Edward Arnold : "The Infant, Nutrition and Management." By Eric Pritchard, M.A., M.D. 3s. 6d. net. "Mentally Defective Children." By A. Binet and Th. Simon, M.D. Translated by W. B. Drummond, M.B. 2s. 6d. net.

From the Author : "Our City Slums : the Experiences of a Medical Practitioner." By H. E. Jones, Ibrox, Glasgow. 6d. net.

Constable and Co., Ltd. : "On Pharmaco-Therapy and Preventive Inoculation Applied to Pneumonia in the African Native." By Sir Almroth E. Wright, M.D., F.R.S. 4s. 6d. net.

Kegan Paul, Trench, Trübner and Co., Ltd. : "Mediterranean Winter Resorts." By E. Reynolds-Ball, F.R.G.S. 5s. net.

T. Werner Laurie, Ltd. : "The Secrets of the German War Office." By Dr. Armgaard Karl Graves. 2s. net.

Longmans and Co. : "Quain's Elements of Anatomy." Eleventh Edition. Vol. II., Part II., Splanchnology." By J. Symington, M.D., F.R.S. 10s. 6d. net.

The Encyclopædia Britannica Co., Limited : Britannic Books for the War—"France"; "Germany"; "Austria-Hungary and Poland"; "Russia and the Balkan States"; "Belgium, Switzerland, and Italy"; "Wars of the Nineteenth Century." 2s. 6d. net per vol., or six vols. 12s. 6d. net.

J. B. Lippincott Co. : "Practical Bandaging." By E. L. Eliason, A.B., M.D. 6s. net.

### Institutional and Public Reports.

New Zealand : Report on Public Health and Hospitals and Charitable Aid for the Year 1913-14.

Wellingborough Cottage Hospital : Annual Report, 1914.

James Murray's Royal Asylum, Perth : Eighty-seventh Annual Report.

Dorset County Asylum : Eighty-second Annual Report.

Glasgow District Mental Hospital, Gartloch : Report for 1913-14.

Wilts County Asylum : Annual Report for Year ended March 31, 1914.

Down District Lunatic Asylum, Downpatrick : Forty-fourth Annual Report.

Rates of Subscription: Twelve months, 6/6; Six months, 4/-; Three months, 2/-, post free to any address in the United Kingdom. Abroad, Twelve months, 8/8; Six months, 5/-; Three months, 2/6, post free.—Address The Subscription Dept., "The Hospital," The Hospital Building, 28/29 Southampton Street, Strand, London W.C.

